# Impacts of Medications on Microbiome-mediated Protection against Enteric Pathogens

**DOI:** 10.21203/rs.3.rs-5199936/v1

**Published:** 2024-10-18

**Authors:** Aman Kumar, Ruizheng Sun, Bettina Habib, Natasha A. Bencivenga-Barry, Ivaylo I. Ivanov, Robyn Tamblyn, Andrew L. Goodman

**Affiliations:** 1 Department of Microbial Pathogenesis and Microbial Sciences Institute, Yale University School of Medicine, New Haven, CT, USA; 2 Department of General Surgery, Xiangya Hospital, Central South University, Changsha, Hunan, China; 3 Clinical and Health Informatics Research Group, McGill University, Montreal, Canada; 4 Department of Microbiology and Immunology, Vagelos College of Physicians and Surgeons, Columbia University, New York, NY, USA; 5 Columbia University Digestive and Liver Diseases Research Center, Vagelos College of Physicians and Surgeons, Columbia University, New York, NY, USA; 6 Department of Epidemiology, Biostatistics and Occupational Health, McGill University, Montreal, Canada; 7 Department of Medicine, McGill University Health Center, Montreal, Canada.

## Abstract

The majority of people in the U.S. manage health through at least one prescription drug. Drugs classified as non-antibiotics can adversely affect the gut microbiome and disrupt intestinal homeostasis. Here, we identified medications associated with an increased risk of GI infections across a population cohort of more than 1 million individuals monitored over 15 years. Notably, the cardiac glycoside digoxin and other drugs identified in this epidemiological study are sufficient to alter microbiome composition and risk of *Salmonella enterica* subsp. Typhimurium (*S*. Tm) infection in mice. The impact of digoxin treatment on *S.* Tm infection is transmissible via the microbiome, and characterization of this interaction highlights a digoxin-responsive β-defensin that alters microbiome composition and consequent immune surveillance of the invading pathogen. Combining epidemiological and experimental approaches thus provides an opportunity to uncover drug-host-microbiome-pathogen interactions that increase infection risk in humans.

## Introduction

Gut microbiome disruption is linked to a wide range of consequences depending on the individual and the environment, including inflammatory bowel diseases^1^, autoinflammatory disease^2,3^, colon cancer^1,4^, and gastrointestinal (GI) infections^5,6^. However, these diseases are individually rare, and the specific triggers and resulting microbiome changes that alter risk in susceptible individuals remain largely unknown. Large-scale, prospective epidemiological studies (of tens to hundreds of thousands of individuals) are a critical strategy to identify risk factors for rare pathologies in human populations, but this approach is challenging in the context of the gut microbiome because available cohorts that are sufficiently powered to include rare events do not include microbiome data captured before and after the onset of disease.

One of the most widespread consequences of microbiome disruption is the loss of colonization resistance in the GI tract, wherein the gut microbiome loses its ability to work in concert with the host immune system to prevent the expansion of pathogens in the gut. Notably, the use of prescription medications is commonly associated with GI side effects, including infections^7–9^. These side effects contribute to drug-related hospitalizations and can limit drug tolerance^10^. Although both oral and non-oral medical drugs can alter microbiome composition^6,11–16^ and gut microbes can metabolize medical drugs^17–19^, potential connections between non-antibiotic prescription drug use, microbiome disruption, and infection risk are largely unknown. Given that the majority of the U.S. population maintains health through the use of one or more prescription medications and diarrheal diseases kill over 1 million people each year^19–23^, such interactions have widespread implications.

Although microbiome data is not directly available in large-scale, longitudinal epidemiological datasets, we reasoned that clinical diagnosis codes reporting GI infection could serve as an indirect indicator of microbiome disruption in a large human population. In this report, we analyzed prescription drug use and clinical diagnosis codes from over 1 million individuals over 15 years to identify drugs associated with an elevated risk of GI infection. We determined that many of these drugs, including the cardiac glycoside digoxin, alter gut microbiome composition and colonization resistance in a mouse model of *Salmonella enterica* subsp. Typhimurium (*S*. Tm) infection. We demonstrate that digoxin induces RORγt-dependent expression of a previously uncharacterized family of enteric β-defensins, which in turn selectively decreases commensal microbes that promote immune surveillance against enteropathogens. Integrating epidemiological and experimental approaches thus provides a strategy to identify triggers of microbiome disruption in human populations that alter the risk for pathologies recorded in clinical diagnosis codes.

## Results

### Identification of prescription drugs that increase infection risk in a large human population.

**Extended Fig. 1|:**
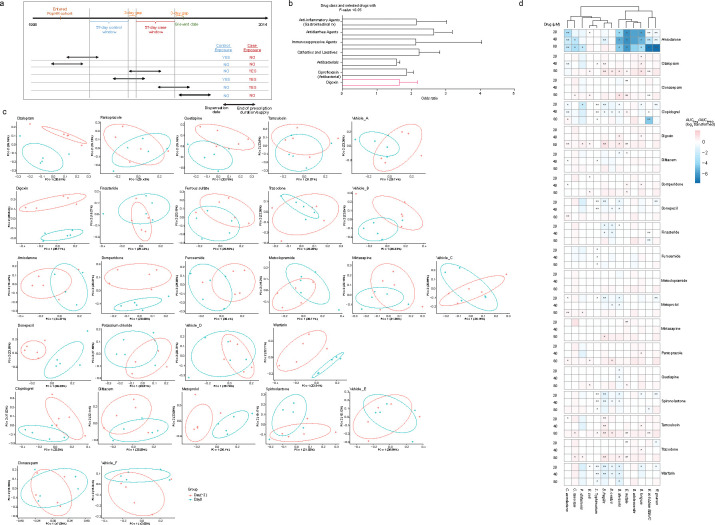
Impact of medications identified in the epidemiological screen on gut microbes in mice and *in vitro*. **a,** Details of the epidemiology study design. Examples of drug exposures that are included or excluded from case or control windows are shown. **b,** Infection risk odds ratio for Digoxin is comparable to the odds ratio measured for drug classes and individual drugs expected to increase infection risk. Error bars represent a 95% confidence interval. **c,** Principal coordinate analysis (PCoA) of Bray-Curtis distances between 16S rRNA sequencing results from fecal samples from C57BL/6N mice before (red) and after treatment (blue). The ellipses in each PCoA plot depicts the 68% confidence marginal relationships among variables in each group generated by an integrated function in the R package “ggplot2”. Each point within the same color represents an individual mouse. **d,** Area under the curve (AUC) comparison for growth of representative bacterial taxa under increasing drug concentrations (20, 40, and 80μM). The tree represents the clustering of taxa based on growth inhibition profiles across all tested drugs. Color shading represents normalized growth (AUC relative to DMSO control). *P< 0.05, **P<0.01.

**Fig. 1|:**
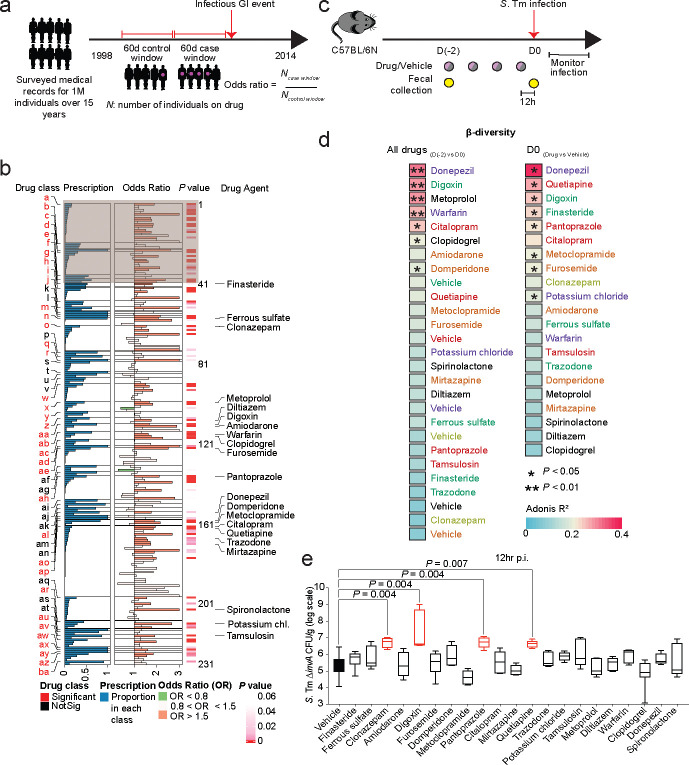
Analysis of 1M individuals over 15 years identifies drugs that increase infection risk in humans and mice. **a,** Design of a case-crossover epidemiological study to identify associations between prescription medications and infectious gastrointestinal (GI) events across >1M individuals. Case and control windows are defined relative to infectious GI events; for each drug, an odds ratio is calculated as the number of individuals (N) taking the drug in case periods relative to control periods. **b,** Epidemiological study results. 21 drugs (with names listed) were identified for further study based on the number of prescriptions within each class, odds ratio > 1.5, and *P*-value <0.05. Letters (a-ba) indicate drug classes, and numbers (1–231) indicate individual drugs (Extended Data Table 2). Drug classes expected to be associated with infection risk (anti-microbial agents, immunosuppressants, antidiarrheals, analgesics and antipyretics, antiemetics, cathartics, and laxatives) are indicated in the shaded area. **c,** Experimental design to study colonization resistance in mice. **d,** Drug-dependent microbiome compositional differences measured using principal coordinate analysis on Bray-Curtis dissimilarity before and after drug treatment (left panel) and between drug- and control-treated animals in the same cohort (right panel). Permutational Multivariate Analysis of Variance (PERMANOVA) analysis was used to calculate the amount of variation. The effect size (R-squared) explains the magnitude of dissimilarities between groups. Drug and vehicle names are colored by mouse cohort. **e,** Multiple drugs identified in the epidemiological screen impact *S.* Tm Δ*invA* pathogen burden in mice. Statistical significance is calculated using the non-parametric Kruskal-Wallis test followed by Dunn’s multiple comparisons test (n=5 mice/group; n=25 mice for vehicle group).

To identify drugs associated with increased infection risk in humans, we examined medical services, hospitalization, and pharmacy claims data within a dynamic cohort of over one million anonymized individuals spanning over 15 years. We conducted a case-crossover study design^24–26^ to perform this analysis, which allowed us to use each case as their own control, effectively adjusting for various known and unknown individual characteristics that could influence the underlying risk of GI infection (Fig. 1a)^24,27^. To this end, we identified the first recorded infectious GI event (indicated by relevant International Classification of Disease, Ninth Revision and Tenth Revision (ICD-9 and ICD-10) codes) for each individual within the 15-year time period (Extended Data Table 1). Next, we established a ~60-day case window immediately preceding the infectious GI event and a ~60-day control window immediately prior to the case window and assessed exposure to prescription drugs in both case and control windows (Extended Data Fig. 1a; see methods for details). Utilizing conditional logistic regression, we identified drug classes that had higher odds of having been dispensed in case windows compared to control windows, indicating an association between the drug classes and the risk of GI infection. This analysis identified several expected drug classes, such as antibacterials, immunosuppressants, and antidiarrheals, suggesting that the case-crossover strategy can successfully identify drug classes that increase GI infection risk (Extended Data Fig. 1b). Notably, this approach also highlights specific non-antibiotic drug classes that increase GI infection disease risk to a similar or greater extent as observed with the expected drug classes (Fig. 1b, Extended Data Table 2). Next, we performed an unbiased analysis of individual drugs from both significant and non-significant drug classes and identified several drugs that were individually associated with increased infection risk. We selected medications meeting specific criteria within each class (prescription number >100, odds ratio >1.5, *P*-value <0.05) and identified 21 candidate drugs for further studies in mice (Fig. 1b, Extended Data Table 2, 3).

### Mouse studies highlight prescription medications that disrupt the microbiome and increase GI infection risk.

We next examined the impact of the 21 drugs identified in the epidemiological study on microbiome composition and infection risk in mice challenged with the model enteropathogen *Salmonella enterica* subsp. Typhimurium (*S*. Tm). Colonization resistance is a critical factor in determining pathogen infection in this model, and most studies rely on streptomycin treatment to disrupt the microbiome prior to infection^28,29^. We administered each of the 21 drugs (Extended Data Table 3), plus vehicle controls in each mouse cohort, twice daily to separate groups of conventional (CV) C57BL/6N mice (Taconic Biosciences) for two days with fecal sampling for 16S rRNA sequencing before treatment initiation and 12 hours after the final dose. WT *S*. Tm induces widespread gut inflammation via the activity of its Type III secretion system (T3SS)-1^30^; because this inflammation further disrupts the microbiome, we conducted the initial drug screen using an *S.* Tm Δ*invA* strain, which lacks an integral structural component of T3SS-1^31^. After a 12-hour washout period following the final drug dose, we infected each animal with *S.* Tm Δ*invA* and monitored infection over time (Fig. 1c). To quantify the impact of drug treatment on the gut microbiome, we evaluated beta diversity within groups before and after drug treatment and between drug- and control-treated groups from the same mouse cohort. Several drugs induced significant compositional changes in the fecal microbiome in at least one of these comparisons (Fig. 1d; Extended Data Fig. 1c); digoxin and donepezil significantly altered microbiome composition in both comparisons. Additionally, four drugs (digoxin, clonazepam, pantoprazole, and quetiapine) significantly increased pathogen burden in mice 12 hours post-infection (Fig. 1e). These drugs did not exhibit widespread antibiotic activity when tested on representative human gut bacterial isolates (Extended Data Fig. 1d).

### Digoxin disrupts colonization resistance.

**Extended Fig. 2|:**
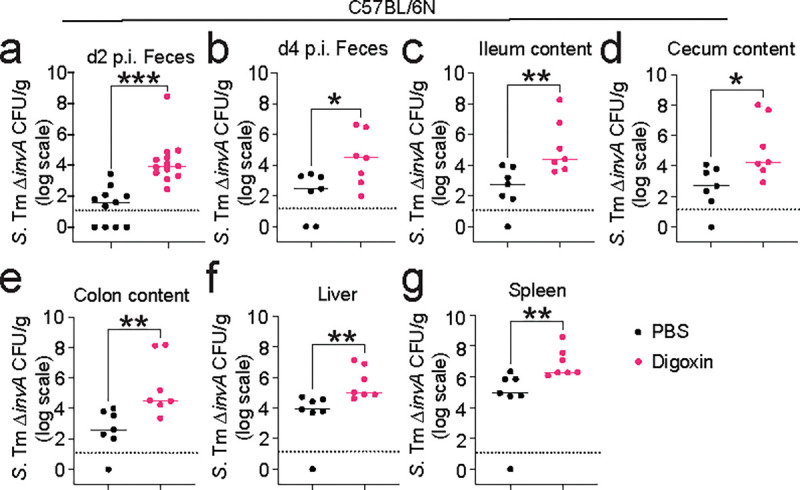
Digoxin pretreatment prior to *S.* Tm Δ*invA* infection leads to increased pathogen colonization and dissemination. **a-g,** Digoxin or PBS-pretreated CV C57BL/6N mice were infected with 10^8^ CFUs of *S.* Tm Δ*invA* 12 h after the final drug or vehicle dose and infection monitored over time. **a,** Pathogen burden in feces at d2 p.i.. **b,** Fecal pathogen burden at d4 p.i.. Mice were sacrificed at d4 p.i. and pathogen burden was enumerated from the ileum (**c**), cecum (**d**), and colon (**e**) contents. Dissemination of *S.* Tm Δ*invA* to extraintestinal tissues was measured in the liver (**f**) and spleen (**g**). A non-parametric Mann-Whitney test is used to compare two groups. Each point represents one mouse. *P<0.05, **P<0.01, ***P<0.001.

**Extended Fig. 3|:**
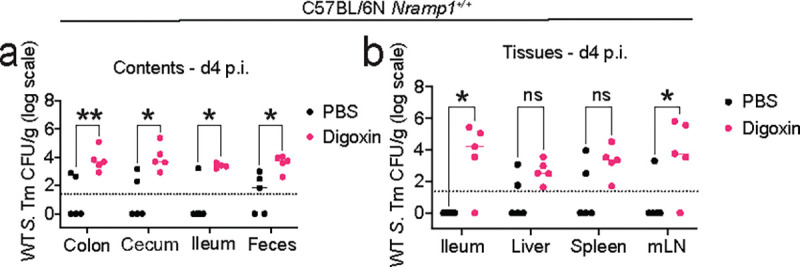
Digoxin pretreatment prior to infection with WT *S.* Tm leads to increased pathogen burden in *Nramp1*^*+/+*^ mice. **a-b,** CV C57BL/6N *Nramp1*^*+/+*^ mice were treated with digoxin or PBS for two days as shown in Fig. 1c; 12h after the final drug or PBS treatment, animals were infected with 10^8^ CFUs of WT *S*. Tm and pathogen burden measured at d4 p.i. in gastrointestinal contents (**a**) and tissues (**b**). A non-parametric Mann-Whitney test is used to compare two groups. Bonferroni-Dunn method is used for multiple comparisons. Each point represents one mouse. *P<0.05, **P<0.01, ns – not significant.

**Fig. 2|:**
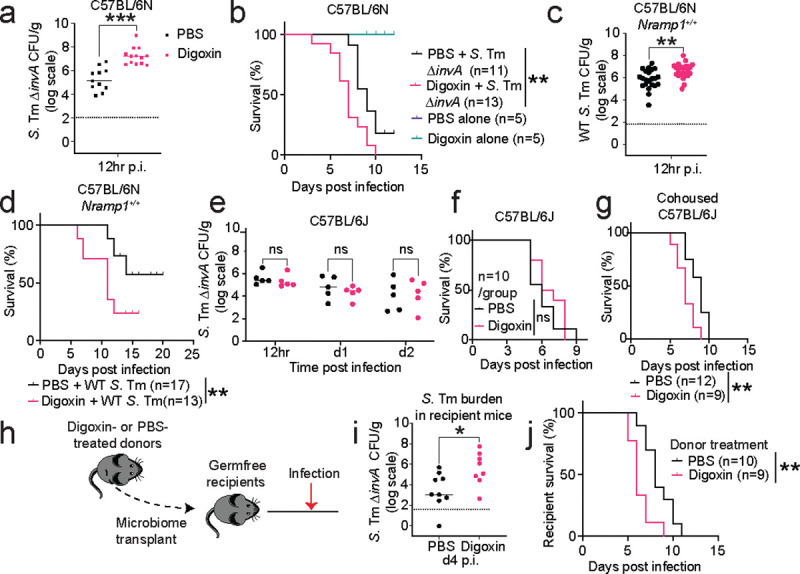
The impact of digoxin on infection risk is transmissible via the microbiome. **a, b,** CV C57BL/6N mice were orally gavaged with digoxin (5mg/kg) or PBS (in 5% dimethyl sulfoxide, DMSO) and infected with *S*. Tm Δ*invA* as in Fig. 1c. **a,** Fecal pathogen burden in PBS-pretreated or digoxin-pretreated mice 12 hr post-infection (p.i.). **b,** Mortality of PBS-pretreated or digoxin-pretreated C57BL/6N mice after *S.* Tm Δ*invA* infection or mock infection. **c,** Pathogen burden in PBS-pretreated or digoxin-pretreated C57BL/6N *Nramp1*^*+/+*^ mice 12 hr after infection with WT *S.* Tm. **d,** Survival of PBS-pretreated or digoxin-pretreated C57BL/6N *Nramp1*^*+/+*^ mice after infection with WT *S.* Tm. **e-f,** Impact of digoxin on infection of CV C57BL/6J mice. Fecal pathogen burden (**e**) and mortality (**f**) in non-cohoused, PBS-treated or digoxin-pretreated C57BL/6J mice after *S.* Tm Δ*invA* infection is shown. **g,** Impact of PBS-pretreatment or digoxin-pretreatment on *S.* Tm infection in C57BL6/J mice that were cohoused with C57BL/6N mice for 14 days. Animals were separated prior to PBS or drug administration and infection. **h,** Schematic of gut microbiome transplantation experiments. **i-j,** Impact of transplantation of gastrointestinal contents from PBS-pretreated or digoxin-pretreated donor mice on *S.* Tm pathogen burden (**i**) and mortality (**j**) in ex-GF recipient mice colonized with either microbiome prior to infection. In **a**, **c**, **e**, **i**, each data point represents one mouse. Bar represents median values, and dotted lines represent the limit of detection. A non-parametric Mann-Whitney test was used for comparison between two groups. In **b**, **d**, **f**, **g**, **j**, the *P-*value was calculated using the Gehan-Breslow-Wilcoxon test. n represents the number of mice. *P<0.05, ** *P*<0.01, ***P<0.001.

We focused on digoxin for further study. In the human cohort, multivariate models indicate that the impact of digoxin on infection risk is independent from concurrent antibiotic, immunosuppressant, and antidiarrheal use (Extended Data Table 4). Additionally, independent replication of *S.* Tm Δ*invA* infection in digoxin-pretreated mice confirmed the significant increase in pathogen burden in fecal samples collected 12 hours, 2 days, and 4 days post infection, as well as in gastrointestinal contents and extraintestinal tissues 4 days post infection (Fig. 2a, Extended Data Fig. 2). Consistent with this increased pathogen burden, digoxin-pretreated mice exhibited significantly increased mortality compared to control mice treated with PBS prior to *S.* Tm Δ*invA* infection (Fig. 2b). C57BL/6N mice rapidly succumb to infection by wildtype (WT) *S.* Tm because they encode a nonfunctional allele of *Nramp1*, which is essential for the bactericidal activity of macrophages; C57BL/6N *Nramp1*^*+/+*^ mice exhibit increased resistance to WT *S.* Tm^32^. Pretreatment of C57BL/6N *Nramp1*^*+/+*^ mice with digoxin prior to infection with WT *S.* Tm increases pathogen burden and mortality in these animals compared to controls treated with PBS prior to WT *S.* Tm infection (Fig. 2c–d, Extended Data Fig. 3). Together, these results suggest that digoxin pretreatment is associated with increased infection risk in humans independent of concurrent use of other drugs and is sufficient to increase infection risk in two mouse models of *S.* Tm infection.

**Extended Fig. 4|:**
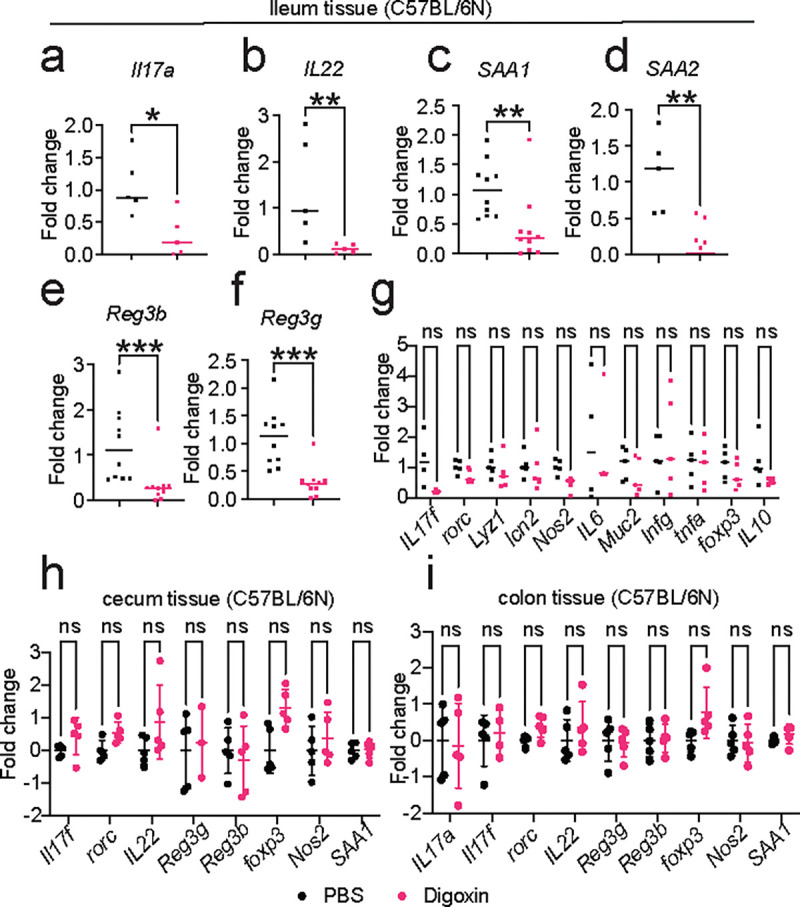
Digoxin pretreatment decreases ileal proinflammatory responses. CV C57BL/6N mice were treated with digoxin or PBS for two days. Mice were sacrificed 12 h after the final drug or vehicle dose, and tissues were collected for gene expression measurement by qRT-PCR. **a-f,** Ileal expression of Th17-related proinflammatory marker genes *IL17a* (**a**), *IL22* (**b**), *SAA1* (**c**), *SAA2* (**d**), *Reg3b* (**e**), and *Reg3g* (**f**). **g,** Ileal expression of other inflammatory marker genes. **h-i**, Expression of representative inflammatory marker genes in cecal (**h**) and colon (**i**) tissue. Fold change is measured relative to *Gapdh* expression. A non-parametric Mann-Whitney test is used to compare two groups. Bonferroni-Dunn method is used for multiple comparisons. Each point is one mouse. *P<0.05, **P<0.01, ***P<0.001, ns – not significant.

Digoxin can alter the activity of retinoic acid-related orphan receptor (RORγt encoded by the *Rorc* gene)^33–35^ . RORγt is essential for the differentiation of naïve T cells into proinflammatory helper T (Th17) cells that produce interleukin-17 (IL-17) and interleukin-22 (IL-22) cytokines^33,34^; other cell types (*e.g.* group 3 innate lymphoid cells (ILC3)) also produce these cytokines in a RORγt dependent manner^36^. These cytokines act on epithelial cells to upregulate the expression of anti-microbial lectins such as *Reg3g* and *Reg3b* and promote neutrophil recruitment to the mucosal surface^37–39^. This orchestrated innate immune response contributes significantly to the establishment of a gut barrier, which is vital for sustaining gut-immune homeostasis and conferring protection against enteric pathogens, and the presence of these factors in the ileum is implicated in resistance against *S*. Tm colonization^37,40^. Notably, expression of IL-17a, IL-22, and IL-17/22-dependent anti-microbial lectins such as *Reg3b* and *Reg3g* was downregulated in digoxin-pretreated mice, specifically in ileal tissue but not in the cecum and colon (Extended Data Fig. 4).

**Extended Fig. 5|:**
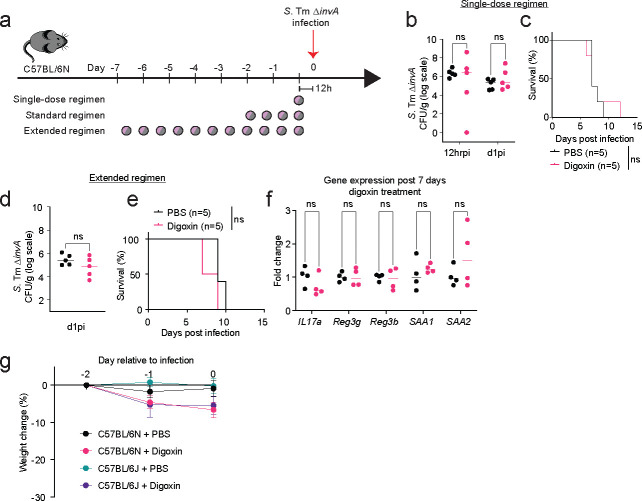
Impact of digoxin pretreatment duration on *S.* Tm infection. **a,** Experimental design. CV C57BL/6N mice were treated with digoxin or PBS for one dose 2hr prior to infection (single dose regimen), twice daily for 2 days followed by a 12-hour washout period (standard regimen), or twice daily for 7 days followed by a 12-hour washout period (extended regimen). Mice were then infected with 10^8^ CFUs of *S*. Tm Δ*invA* and infection monitored over time. **b-c,** Pathogen burden at 12hr p.i. (**b**) and mortality (**c**) after single-dose drug or control treatment. **d-f,** Pathogen burden at 12hr p.i. (**d**), mortality (**e**), and expression of proinflammatory marker genes in ileum tissue (**f**) after the extended regimen drug or control treatment. **g,** Impact of digoxin or PBS treatment on the weight of CV C57BL/6N and C57BL/6J mice. In **f**, fold change is measured relative to the mouse housekeeping gene, *Gapdh*. In **b, d, f,** a non-parametric Mann-Whitney test is used to compare two groups. For survival analysis, the Gehan-Breslow-Wilcoxon test is used. ns – not significant.

We next assessed whether the direct effect of digoxin on the host is likely to explain the differences in susceptibility to infection outcome irrespective of the effect of digoxin on the microbiome. To this end, we first determined whether other digoxin treatment regimens altered the risk of infection. We assessed *S.* Tm Δ*invA* infection in C57BL/6N mice treated with a single dose of digoxin (2 hours before infection) or given an extended treatment regimen (twice-daily treatment for 7 days, prior to a 12-hour washout before infection) (Extended Data Fig. 5a). Unlike the 2-day treatment group, neither of these treatment strategies altered infection outcome relative to control animals treated with PBS prior to infection (Extended Data Fig. 5b–e). Consistent with this result, proinflammatory *S*. Tm resistance genes that were significantly downregulated after 2 days of digoxin treatment returned to baseline levels after 7 days of drug administration (Extended Data Fig. 5f). These distinct phenotypes, depending on the timing of digoxin treatment, suggest that the impact of digoxin on the host may not be sufficient to explain the increased *S*. Tm susceptibility.

**Extended Fig. 6|:**
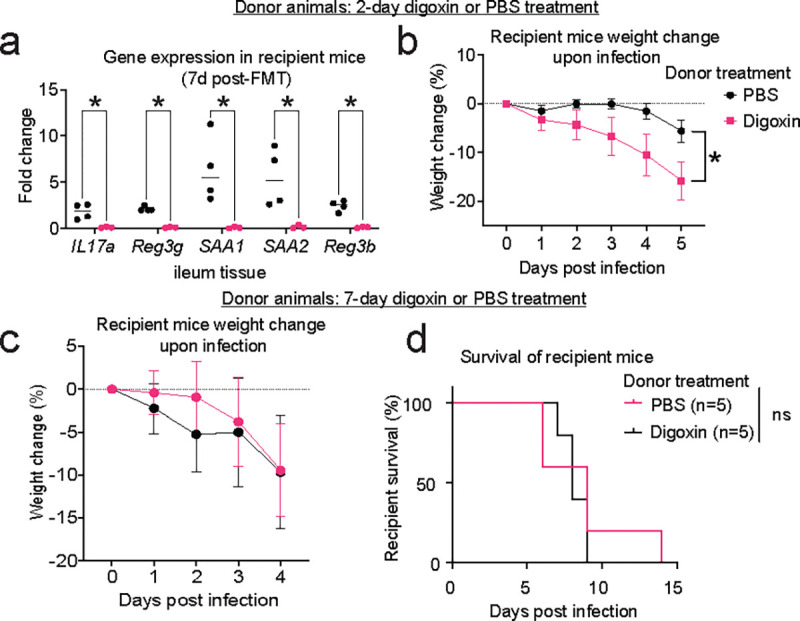
Altered immune responses and pathogen susceptibility are microbiome dependent. **a-b,** Characterization of recipient mice after transplantation of gut microbiomes from donor animals treated with digoxin or PBS for 2 days (standard regimen). **a,** Gene expression in ileal tissue of recipient mice as measured by qRT-PCR. **b,** Weight of recipient mice after infection with *S.* Tm ∆*invA.* **c-d,** Characterization of recipient mice after transplantation of gut microbiomes from donor animals treated with digoxin or PBS for 7 days (extended regimen). **c,** Weight of recipient mice after infection with *S.* Tm ∆*invA.* **d,** Survival of recipient mice after infection with *S.* Tm ∆*invA.* In **a**, fold change is measured relative to the mouse housekeeping gene, *Gapdh*. In **a**, **b**, a non-parametric Mann-Whitney test is used to compare two groups. In **a,** multiple comparisons are made using the Bonferroni-Dunn method. For survival analysis, the Gehan-Breslow-Wilcoxon test is used. * *P*< 0.05, ns – not significant.

Because digoxin treatment alters microbiome composition (Fig. 1d, Extended Data Fig. 1c), we next examined whether mice with different microbiomes exhibit similar digoxin responses. While both C57BL/6N mice and C57BL/6J mice (obtained from Jackson Laboratories) exhibited a slight reduction in weight upon digoxin treatment, pretreatment with the drug did not cause increased pathogen burden or altered mortality in C57BL/6J mice infected with *S.* Tm ∆*invA* (Fig. 2e–f, Extended Data Fig. 5g). Cohousing these animals with Taconic (C57BL/6N) mice for two weeks to allow for microbiome exchange altered this phenotype: C57BL/6J mice cohoused with C57BL/6N mice were significantly more susceptible to infection after digoxin pre-treatment compared to cohoused controls that were not treated with digoxin prior to infection (Fig. 2f–g). To determine whether the impact of digoxin on pathogen susceptibility is transmissible via the microbiome, we next treated C57BL/6N donor animals with digoxin or PBS for 2 days as above; after a 12-hour washout period, we transplanted the gut contents of each donor animal into a germ-free (GF) recipient mouse. Seven days after microbiome transplantation, recipient mice were sacrificed (to measure proinflammatory markers) or infected with *S.* Tm Δ*invA* (Fig. 2h). Recipient mice carrying the microbiomes of digoxin-treated donor animals exhibited significantly reduced expression of proinflammatory markers prior to infection and increased pathogen burden, weight loss, and mortality after infection compared to recipient animals colonized with the microbiomes of PBS-treated animals (Fig. 2i–j, Extended Data Fig. 6a–b). By contrast, transplantation of gut contents from C57BL/6N donor animals treated for 7 days with digoxin or PBS and the same 12-hour washout period, into GF recipient mice resulted in no difference in *S.* Tm Δ*invA* susceptibility between recipient groups (Extended Data Fig. 6c–d). Together, these data suggest that the direct interaction between digoxin and host cells (including any trace carryover from microbiome transplantation after the 12-hour washout in donor animals and 7 days in recipient animals) is not sufficient to increase susceptibility to *S*. Tm infection.

### Digoxin-mediated depletion of immunomodulatory gut microbes increases susceptibility to S. Tm infection.

**Extended Fig. 7|:**
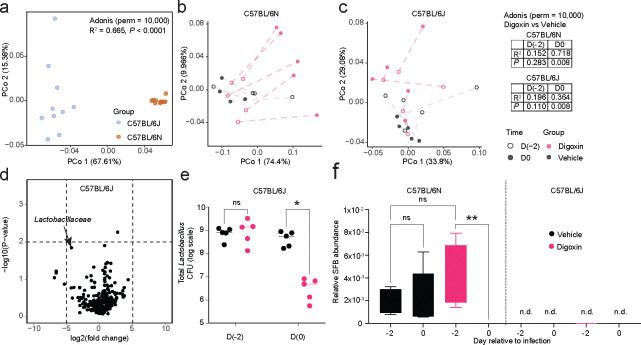
Impact of digoxin treatment on the mouse microbiome. **a**, Principal coordinate analysis (PCoA) using weighted UniFrac distance matrices were used to calculate the compositional differences between untreated C57BL/6N and C57BL/6J mice. **b-c,** PCoA plots using weighted UniFrac distance matrices were used to compare the compositional change of untreated, digoxin-treated, or PBS-treated fecal samples in **b,** C57BL/6N, and **c,** C57BL/6J mice. In **a-c,** Permutational Multivariate Analysis of Variance (PERMANOVA) analysis using the adonis function with 10,000 permutations was used to calculate the amount of variation. The effect size (R-squared) explains the magnitude of dissimilarities between groups. **d,** Volcano plot showing differentially abundant taxa in fecal contents in PBS-pretreated and digoxin-pretreated C57BL/6J mice. **e,** Impact of digoxin or PBS treatment (standard regimen) on the abundance of *Lactobacillus sp.* as measured by selective plating on McConkey agar. A non-parametric Mann-Whitney test is used to compare two groups. Multiple comparisons are done using the Bonferroni-Dunn method. **f,** Relative SFB abundance based on 16S rRNA sequencing of fecal samples collected from C57BL/6N and C57BL/6J mice 12h after the final PBS or digoxin dose of a 2-day (standard) treatment regimen. Kruskal-Wallis test was used to compare three or more groups, followed by Dunn’s multiple comparisons test. **a-c, e**, Each data point represents one mouse. * *P*< 0.05, ** *P*< 0.01, ns – not significant, n.d. – not detected.

**Extended Fig. 8|:**
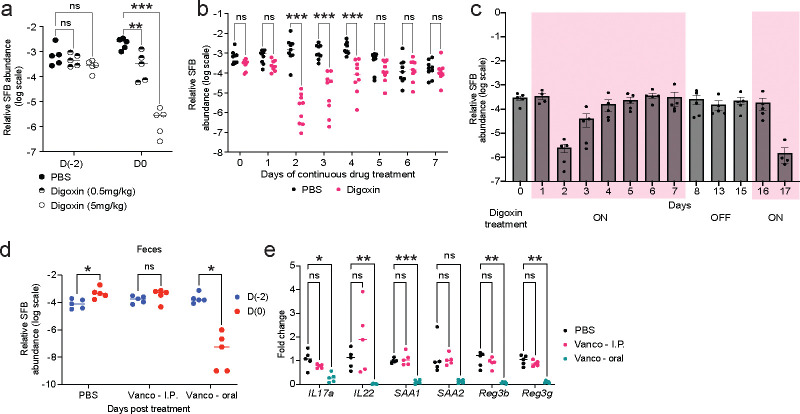
Impact of digoxin dose and treatment duration on SFB abundance in mice. **a,** SFB abundance in PBS-pretreated, digoxin-pretreated (5 mg/kg; standard dose), or digoxin-pretreated (0.5 mg/kg) C57BL/6N mice relative to total bacteria, as measured by qPCR. Samples were collected 12 hours after the last treatment dose. Two-way ANOVA was performed, followed by Dunnett’s multiple comparisons test. **b,** SFB abundance over time in fecal samples from C57BL/6N mice continuously treated (7 days, 2x/day) with PBS or digoxin. Samples were collected 12 hours after the previous treatment dose, and SFB abundance was measured by qPCR and normalized relative to the total bacteria in the sample. **c,** SFB abundance over time in fecal samples from C57BL/6N mice intermittently treated with digoxin. Mice were administered digoxin 2x/day on days 1–7, followed by a 7-day rest period (no treatment); digoxin treatment was resumed (2x/day) on days 16–17. SFB abundance was measured as in (**b**). **d-e**, CV C57BL/6N mice were treated with vancomycin either intraperitoneally or by oral gavage using the standard two-day treatment regimen. PBS was administered intraperitoneally as a control. **d**, SFB abundance. **e**, Expression of select maker genes 12h after the final treatment dose. A non-parametric Kruskal-Wallis test was used to compare three or more groups, followed by Dunn’s multiple comparisons test. In **b**, **d**, a non-parametric Mann-Whitney test was used to compare two groups, followed by multiple comparisons using the Bonferroni-Dunn method. *P< 0.05, ** *P*< 0.01, *** *P*< 0.001, ns – not significant.

**Fig. 3|:**
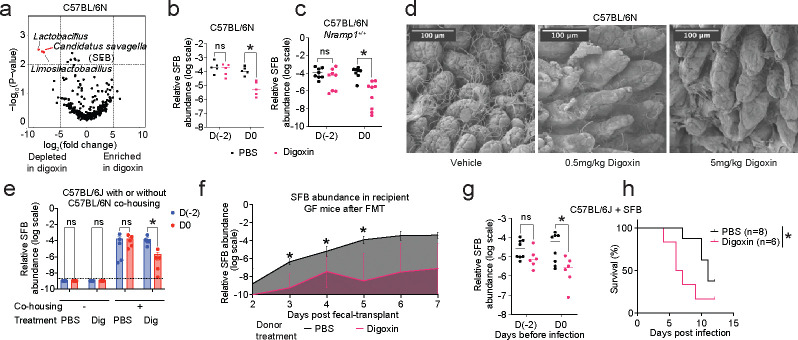
Digoxin-mediated depletion of segmented filamentous bacteria (SFB) increases susceptibility to *S*. Tm infection in mice. **a,** Volcano plot showing differentially abundant taxa in fecal contents in PBS-pretreated and digoxin-pretreated C57BL/6N mice. **b, c,** SFB abundance in PBS-pretreated or digoxin-pretreated C57BL/6N (**b**) and C57BL/6N *Nramp1*^*+/+*^ (**c**) mice relative to total bacteria, as measured by quantitative PCR (qPCR). **d,** Scanning electron microscopy of terminal ileum of mice pretreated with PBS or digoxin. **e,** C57BL/6J mice cohoused with C57BL/6N animals acquire SFB, which is reduced upon digoxin (Dig) treatment. Animals were separated prior to PBS or drug administration. **f,** Relative abundance of SFB in ex-GF recipient mice after transplantation of gastrointestinal contents from PBS-pretreated or digoxin-pretreated C57BL/6N donor animals. **g,h,** SFB colonization is sufficient to alter the response of C57BL/6J mice to digoxin. SFB colonization was conducted on day −14 relative to infection, and drugs were administered for two days before infection as in Fig. 1c. Relative abundance of SFB after PBS or digoxin pretreatment (**g**) and mortality after *S.* Tm Δ*invA* infection (**h**) is shown. The *P-*value was calculated using the Gehan-Breslow-Wilcoxon test. n represents the number of mice. In **b-c** and **e-g,** a non-parametric Mann-Whitney test was used to compare two groups. Multiple comparisons are made using the Bonferroni-Dunn method; * indicates *P*< 0.05.

Because the impact of digoxin on infection was transmissible via the microbiome, we next evaluated digoxin-mediated microbiome changes in C57BL/6N and C57BL/6J mice. Digoxin treatment significantly altered gut microbiome composition in both C57BL/6N and C57BL/6J mice (Extended Data Fig. 7a–c), including decreased relative abundance of multiple *Lactobacillus* taxa (Fig. 3a, Extended Data Fig. 7d–e). Digoxin treatment also led to a marked depletion in *Candidatus savagella* in C57BL/6N mice; this microbe was absent in C57BL/6J animals (Fig. 3a, Extended Data Fig. 7f)^41^. *C. savagella* is a member of the segmented filamentous bacteria (SFB) group of spore-forming, Gram-positive bacteria which have not been successfully cultured under axenic conditions. SFB attaches to the ileal epithelium and triggers a cascade of host immune responses, including upregulation of the serum amyloid A (SAA1/2) pathway^37,42^, activation of RORγt, and results in maintaining a proinflammatory gut environment that protects against invading pathogens^39,43,44^. Consistent with the 16S rRNA sequencing results, targeted (qPCR) measurement of SFB levels in C57BL/6N WT and *Nramp1*^*+/+*^ mice indicated a significant reduction in fecal SFB levels after digoxin pretreatment (Fig. 3b–c). Scanning electron microscopy (SEM) analyses of ileal tissue from digoxin- and vehicle-pretreated mice reveal that digoxin pretreatment nearly completely eliminates SFB from the ileal epithelium (Fig. 3d). Mice pretreated with 0.5 mg/kg digoxin also show significant reduction in SFB as measured by qPCR and SEM, indicating that 2 days of digoxin treatment alters the ability of SFB to attach to the ileal epithelium or colonize the gut across the range of digoxin doses used in the literature^33,34^ (Fig. 3d, Extended Data Fig. 8a).

Three lines of evidence further implicate digoxin control of immunomodulatory microbes such as SFB as a key step in determining the impact of the drug on pathogen susceptibility. First, digoxin treatment of SFB-negative C57BL/6J mice has no impact on *S.* Tm infection; C57BL/6J mice cohoused with C57BL/6N animals become colonized with SFB, which decreases in these C57BL/6J mice upon digoxin treatment (Fig. 3e). Second, SFB levels are significantly reduced in ex-GF recipient mice engrafted with the microbiomes of digoxin-treated donor mice compared to recipient mice engrafted with microbiomes of PBS-treated donors (Fig. 3f). Third, we directly colonized C57BL/6J mice with fecal material from SFB-monoassociated gnotobiotic mice; these animals became robustly colonized and digoxin treatment resulted in a significant depletion in the SFB population (Fig. 3g) and increased infection susceptibility (Fig. 3h). Consistent with the observation that the impact of digoxin on infection susceptibility and proinflammatory marker genes is transient (Extended Data Fig. 5b–f), SFB levels recover in mice treated with digoxin for 5 days or more (Extended Data Fig. 8b). A brief drug discontinuation period is sufficient to render the system digoxin-sensitive again (Extended Data Fig. 8c). Finally, we treated C57BL/6N mice with vancomycin to understand whether treatment with antibiotics that reduce SFB levels is sufficient to recapitulate the impact of digoxin on SFB-dependent proinflammatory marker genes. Indeed, oral vancomycin reduced SFB and expression of these genes (Extended Data Fig. 8d–e). Together, these results suggest that treatments that reduce SFB levels for two days are sufficient to alter gut immune homeostasis.

### Digoxin controls SFB colonization through the induction of a family of β-defensins.

**Extended Fig. 9|:**
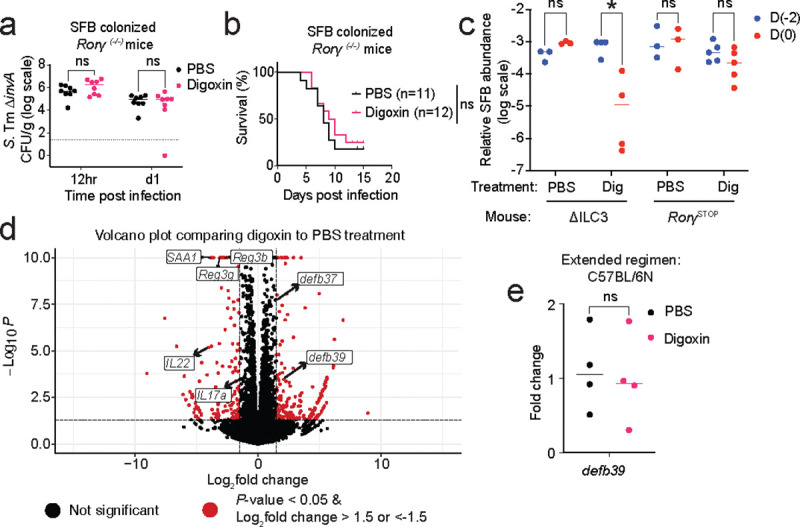
Characterization of the role of RORγt and enteric β-defensins in digoxin response. **a-b,** Impact of digoxin or PBS pretreatment on *S.* Tm ∆*invA* infection in SFB-colonized *Rorγ* ^*−/−*^ mice. Pathogen burden (**a**) and mortality (**b**) is shown. **c,** Impact of PBS- or digoxin-pretreatment on SFB levels in SFB-colonized DILC3 mice and littermate *Rorγ*^STOP^ controls. **d,** Volcano plot of RNA sequencing data showing differentially expressed genes between digoxin-pretreated and PBS-pretreated C57BL/6N mice. **e**, *defb39* expression from ileum tissue of mice pretreated with PBS or digoxin for an extended (7-day) regimen. In **a**, **c, e**, a non-parametric Mann-Whitney test was used to compare the two groups. In **c,** one In **b**, the Gehan-Breslow-Wilcoxon test is used. ** *P*< 0.01, ns – not significant.

**Fig. 4|:**
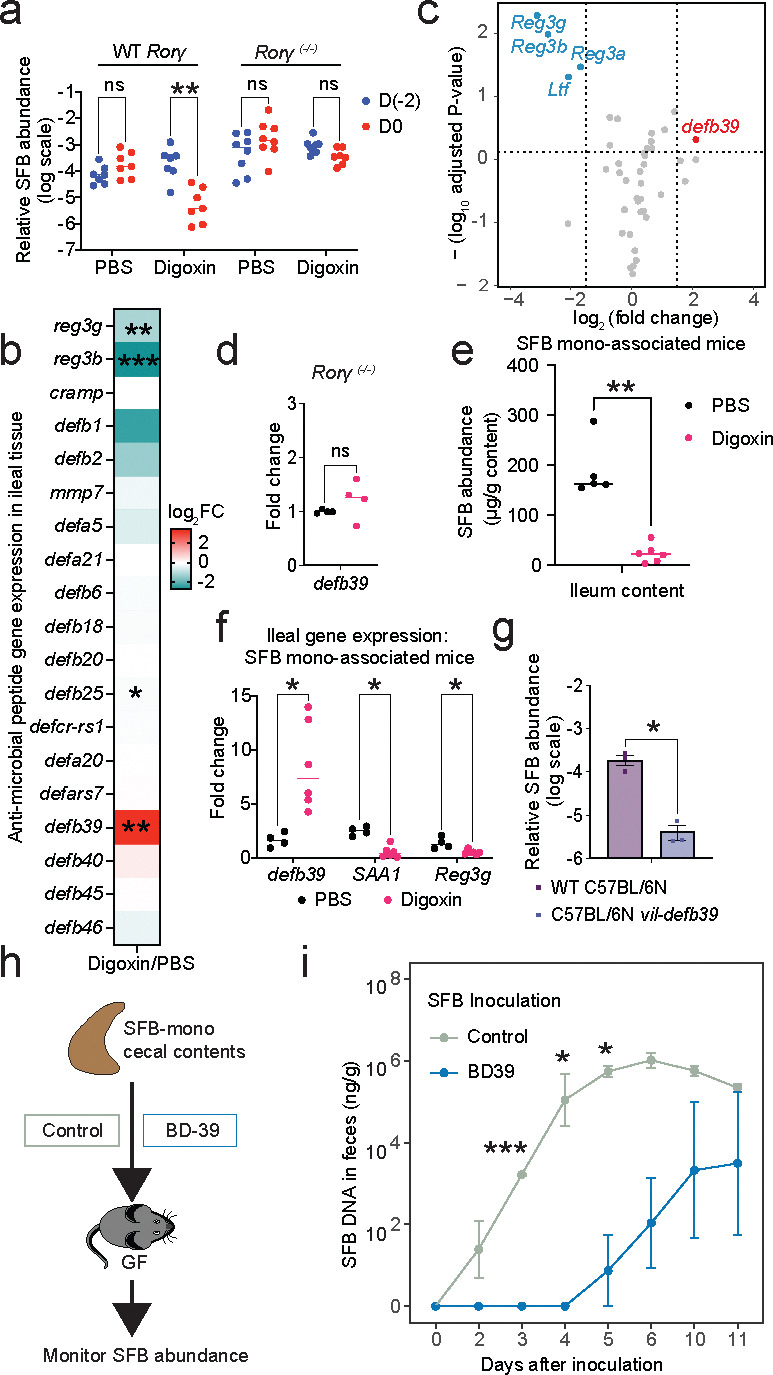
A digoxin-inducible, RORγt-dependent β-defensin controls SFB levels in the mouse gut. **a,** Impact of PBS- or digoxin-pretreatment on SFB levels in SFB-colonized *Rorγ* ^*−/−*^ mice and their WT littermate controls. **b-c,** Relative expression of genes encoding anti-microbial peptides (AMPs) in ileal tissue of C57BL/6N mice with and without digoxin pre-treatment, as measured by qRT-PCR of a targeted gene panel (**b**) and RNA-seq (**c**). Experiments were performed on separate groups of mice, and an unpaired *t* test was used to calculate statistical significance. In **b,** FC indicates fold change. **d,** *defb39* gene expression in ileal tissue of SFB-colonized *Rorγ*^*−/−*^ mice after PBS or digoxin pretreatment. **e,** Absolute SFB abundance in the ileum content of ex-GF mice mono-colonized with SFB and treated with PBS or digoxin. **f,** Expression of digoxin-induced (*defb39*) and SFB-responsive (*SAA1, Reg3g*) genes in ileal tissue of ex-GF mice mono-colonized with SFB and treated with PBS or digoxin. **g,** SFB abundance in fecal samples from *vil-defb39* transgenic mice and WT controls. **h,** Schematic for measuring antimicrobial activity of purified BD-39 against SFB. **i,** Absolute SFB abundance over time in ex-GF mice colonized with BD-39 incubated samples or buffer control-incubated samples. A two-sample Welch t-test was used to compare the two groups. In **b, d, f,** fold change is measured relative to the mouse housekeeping gene, *Gapdh*. In **a, d, e, f, g,** a Mann-Whitney test is used to compare two groups. ns, not significant; *P<0.05, **P<0.01., ***P<0.001.

Because digoxin binds the master transcription factor RORγt^33^, we next examined whether the digoxin-dependent decrease in SFB is RORγt dependent. Digoxin pretreatment of SFB-colonized *Rorγ* ^*−/−*^ mice^45^ did not result in reduced SFB levels or increased susceptibility to infection (Fig. 4a, Extended Data Fig. 9a–b), suggesting that 1) digoxin does not directly kill SFB; and 2) digoxin reduces SFB levels in a RORγt-dependent manner. Although RORγt is expressed in both Th17 and ILC3 cells, mice specifically lacking ILC3 cells^46^ exhibit SFB reduction in response to digoxin as observed in wildtype animals, while mice lacking both Th17 and ILC3 cells are insensitive (Extended Data Fig. 9c). This suggests that digoxin can decrease SFB levels independently of ILC3 cells. Because anti-microbial peptides (AMPs) such as *Reg3g* and α-defensins are linked with reduced SFB abundance in the gut^47,48^, we measured the expression of a panel of AMPs in ileal tissue of digoxin- and PBS-pretreated C57BL/6N mice. Digoxin pretreatment generally had no effect or resulted in downregulation of most of the measured AMPs; however, drug treatment resulted in significant upregulation of the gene encoding β-defensin 39 (BD-39) (Fig. 4b). RNA sequencing of ileal tissue (from a separate cohort of digoxin- and PBS-pretreated C57BL/6N mice) confirmed the digoxin-dependent *defb39* upregulation observed in the targeted gene expression study, and further identified a second member of this β-defensin family (BD-37) as potentially upregulated in digoxin-pretreated compared to PBS-treated animals (Fig. 4c, Extended Data Fig. 9d). Consistent with the digoxin-dependent reduction in SFB levels and prior targeted gene expression measurements, these RNA-seq measurements validated digoxin-induced downregulation of SFB-dependent genes (*SAA1, SAA2*)^37^ as well as Th17/ILC3 pathway proinflammatory factors involved in control of *S.* Tm infection (*IL-17a*, *IL-22*, *Reg3b*, *Reg3g*; Extended Data Fig. 9d).

Notably, digoxin pretreatment of SFB-colonized *Rorγ* ^*−/−*^ mice does not alter *defb39* expression in these animals, and WT mice administered digoxin for an extended (7 day) interval return *defb39* expression to untreated levels (Fig. 4d, Extended Data Fig. 9e). Further, digoxin treatment of SFB-monoassociated GF mice resulted in significantly increased expression of *defb39,* reduced SFB abundance, and decreased expression of canonical SFB-regulated genes (*SAA1*, *Reg3g*) (Fig. 4e–f). Together, these results suggest that other members of the microbiota are not necessary for digoxin-dependent *defb39* upregulation or SFB reduction. We next determined whether the upregulation of *defb39* is sufficient to modulate SFB levels. To this end, we generated transgenic mice that constitutively express *defb39* under the control of the intestinal epithelium-specific Villin (encoded by *Vil1* gene) promoter. Transgenic C57BL/6N *vil-defb39* mice do not exhibit any major pathologies. Notably, transgenic *vil-defb39* expression significantly reduces SFB levels compared to wildtype C57BL/6N controls; indeed, SFB levels in *vil-defb39* animals are similar to digoxin-pretreated, wildtype mice (Fig. 4g). To test whether BD-39 exhibits direct antimicrobial activity against SFB (which cannot be cultured *in vitro*), we expressed and purified BD-39, incubated cecal contents from SFB-monoassociated mice with this purified BD-39 or control buffer, inoculated the incubated samples into GF mice, and measured SFB viability in the inoculum by quantifying SFB levels over time. Notably, SFB levels are significantly reduced in mice colonized with the BD-39 incubated samples as compared to the buffer control-incubated samples, indicating that BD-39 can kill SFB directly (Fig. 4h–i). These data suggest that digoxin pretreatment induces *defb39* expression in a RORγt-dependent manner and that *defb39* expression is sufficient to reduce SFB levels and consequent immune responses in mice.

### Digoxin alters colonization resistance in mice carrying human gut microbiomes.

**Extended Fig. 10|:**
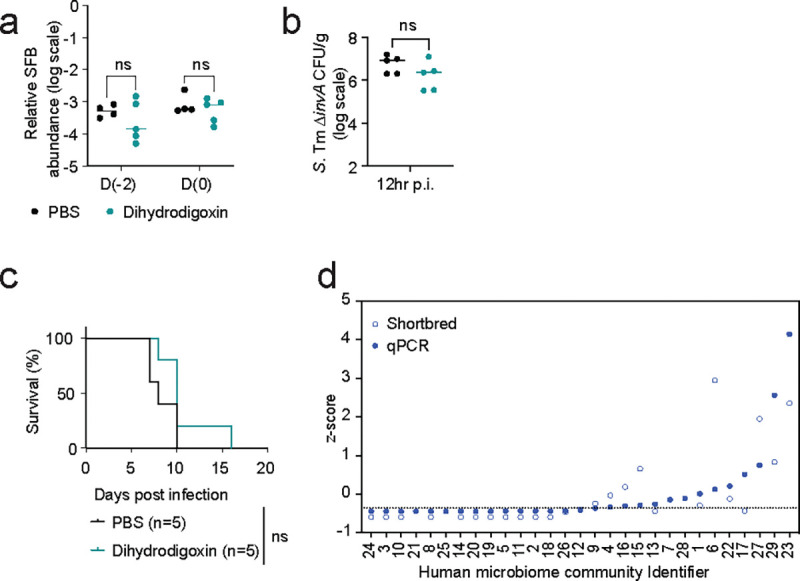
Impact of microbial digoxin metabolites on *S.* Tm ∆*invA* infection. **a-c,** CV C57BL/6N mice treated with dihydrodigoxin (5 mg/kg) or PBS (standard regimen). 12 hours after the final drug or buffer treatment, mice were infected with 10^8^ CFUs of *S*. Tm Δ*invA* and infection monitored over time. **a,** Relative abundance of SFB, normalized to total bacteria, in fecal samples before and after dihydrodigoxin or PBS treatment. **b,** Pathogen burden at 12 hr post infection (p.i.) **c,** Survival curve. **d,** Estimation of *cgr2* gene abundance across 29 fecal communities from unrelated human donors, as measured from metagenomic sequencing and Shortbred analysis or targeted qPCR analysis. In **a**, **b**, a non-parametric Mann-Whitney test was used to compare the two groups. In **c**, the Gehan-Breslow-Wilcoxon test is used. ns - not significant.

**Fig. 5|:**
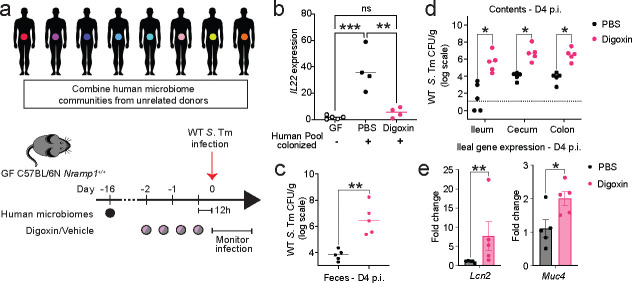
Digoxin increases the susceptibility of gnotobiotic mice colonized with human microbial communities to WT *S*. Tm infection. **a,** Experimental design. Human fecal samples were pooled and used to colonize GF C57BL/6N *Nramp1*^*+/+*^ recipient mice. Mice were pretreated with PBS or digoxin as in Fig. 1c and infected with WT *S.* Tm. **b,** Expression of *IL22* in ileal tissue of GF *Nramp1*^*+/+*^ mice and ex-GF *Nramp1*^*+/+*^ animals colonized with pooled human communities prior to infection. Ileal tissues were collected 12 hours after the final treatment dose. Statistical tests were performed using ordinary one-way ANOVA, followed by Tukey’s multiple comparisons test. **c-d,** Ex-GF *Nramp1*^*+/+*^ mice pretreated with PBS or digoxin were infected with WT *S*. Tm, and pathogen burden in feces (**c**), contents of ileum, cecum, and colon (**d**) is shown. **e,** Gene expression of *S.* Tm-responsive inflammatory marker genes in ileal tissue from mice from (**a**) at day 4 post infection. A non-parametric Mann-Whitney test is used to compare two groups. Each data point is an individual mouse. *P<0.05, **P<0.01, ***P<0.001.

Although SFB is rare in humans^49,50^, individuals exhibit extensive interpersonal variation in other Th17-inducing gut microbes^42,51^. Additionally, human microbiomes exhibit interpersonal variation in digoxin metabolism (inactivation), which is mediated by the microbial enzyme Cgr2^52^. Oral administration of the Cgr2-dependent digoxin metabolite, dihydrodigoxin, does not alter SFB levels or *S*. Tm infection in mice (Extended Data Fig. 10a–c). To establish a human microbiome community that can create a proinflammatory environment in mice but does not inactivate digoxin, we first estimated *cgr2* gene abundance across microbiomes of 30 human donors^17^ using targeted and metagenomic approaches; approximately half of the individuals lacked detectable *cgr2* (Fig. 5a, Extended Data Fig. 10d). We next rederived C57BL/6N *Nramp1*^*+/+*^ mice to the germfree state, colonized these animals with a pooled microbiome community from eight *cgr2-*negative human donors, and measured digoxin response in these animals. Colonization of GF *Nramp1*^*+/+*^ animals with these human communities resulted in robust expression of *IL22* (Fig. 5b). Notably, digoxin pretreatment reduced the expression of *IL22* in the ileum, as observed in CV C57BL/6N (SFB-positive) mice (Fig. 5b, Extended Fig. 4b). Further, infection with WT *S.* Tm resulted in significantly increased pathogen burden in digoxin-treated mice compared to PBS-treated controls carrying the same human community (Fig. 5c–d). Consistent with this increased pathogen burden, digoxin-pretreated, *S.* Tm-infected mice exhibit elevated expression of *S.* Tm-responsive inflammatory marker genes^30,53^ (Fig. 5e). Collectively, these studies suggest that the impact of digoxin pretreatment on infection risk is conserved in the context of SFB-negative human microbiomes.

## Discussion

Multiple GI diseases are linked to the microbiome. Here, we used large-scale epidemiological human data to identify prescription medications that represent risk factors for infectious GI diseases. Nearly half of the non-antibiotic medications identified in the epidemiological study altered microbiome composition, susceptibility to infection, or both in mice. This study focuses on digoxin, which alters infection risk in mice by inducing expression of beta-defensins that reconfigure the gut microbiome, leading to immune reprogramming and ameliorated pathogen response. In the context of a mouse gut microbiome, immune reprogramming is mediated by SFB; gnotobiotic mice colonized with IL-22-inducing human communities lacking SFB exhibit similar responses, suggesting a conserved response across mice and humans.

Three lines of evidence indicate that the gut microbiome plays a key role in digoxin-dependent infection risk. First, transplantation of digoxin-treated microbiomes into GF mice increases infection risk compared to recipient animals colonized with non-treated microbiomes, suggesting that a digoxin-altered microbiome is sufficient to confer altered infection risk in the absence of the drug itself. Second, no difference in infection risk is observed in C57BL/6J mice administered digoxin, or C57BL/6N mice maintained on an extended digoxin treatment regimen, indicating that direct administration of the drug is not sufficient to alter infection risk. Third, the impact of digoxin on proinflammatory gene expression can be recapitulated by alternate means of SFB elimination (*e.g.,* oral vancomycin). While this treatment impacts the levels of multiple taxa, the observation that digoxin induces similar responses in conventional C57BL/6N and gnotobiotic SFB-monoassociated mice suggests that this species alone is sufficient to increase susceptibility to *S.* Tm infection in response to digoxin.

BD-39, an ortholog of human BD-1^54^, is a member of a family of beta-defensins that are conserved across mammals; humans encode more than 30 family members^55^. Human BD-1 is expressed in the gastrointestinal tract, including the colon and ileum, and is induced in the presence of pathogens or other signals^56,57^. While infection and inflammation at the mucosal surface are generally thought to be the triggers for induction of beta-defensins^58^, our data indicate that xenobiotic compounds such as digoxin also serve as inducing signals. Notably, BD-39 expression returns to baseline levels upon extended digoxin treatment. While the specific attenuating mechanism requires further study, induction of regulatory T cells can promote immunogenic tolerance against compounds such as food antigens^59,60^. In mice subjected to an extended digoxin treatment regimen, temporary discontinuation followed by resumption of digoxin treatment re-sensitizes animals to the impact of digoxin on BD-39 expression, SFB levels, and consequent immune responses. The epidemiological study included patients that initiated digoxin treatment at the end of the case window, as well as individuals that were temporarily noncompliant (missed doses), which could explain why digoxin increases infection risk in patients despite attenuation over time in mice. Notably, ~25% of digoxin patients are typically noncompliant due to under-dosing^61^.

Whether digoxin-dependent BD-39 expression and consequent microbiome remodeling is adaptive or accidental remains unclear. Like many medications, digoxin is derived from a poisonous plant (foxglove). Multiple examples of microbiome-mediated response to plant compounds are observed in nature^62^. For example, certain wild populations of desert woodrat (*Neotoma lepida*) persist on creosote, a toxic plant that kills laboratory rats and mice^63–65^. Creosote consumption alters the microbiome of naïve woodrats, and fecal microbiome transplant from creosote-adapted donor animals to creosote-naïve woodrats or to laboratory rats significantly protects these animals from creosote toxicity^63,66^. While the mechanism of creosote-mediated microbiome remodeling is unknown, the host can play a key role in shaping such interactions: for example, 2,3,7,8-tetrachlorodibenzofuran (TCDF), an environmental toxin and ligand for the aryl hydrocarbon receptor (AhR), remodels the microbiome in an AhR-dependent manner^67^. Similarly, dietary changes and the availability of nutrients can remodel the cellular environment of the intestinal epithelia and its interactions with gut lymphocytes^68^. Given that digoxin acts directly on RORγt to modulate various inflammatory pathways, it is possible that the consumption of toxic foxglove plants enables the host to dampen the pre-existing proinflammatory gut environment by eliminating these immunomodulatory microbes.

Multiple factors, including environmental exposures, diet, and pathogen infection, can also disrupt microbiome composition; epidemiological studies track each of these events in large human cohorts^69–73^. Further, medical records document diverse health outcomes with potential links to microbiome disruption, including non-infectious colitis, colon cancer, and drug efficacy and toxicity^74–77^. The combination of epidemiological and experimental approaches described here could be similarly applied to define mechanistic relationships between other causes and consequences of microbiome disruption in humans, and potentially identify individuals at risk as well as therapeutic interventions.

## Extended Data
